# Non-diagnostic time in digital pathology: An empirical study over 10 years

**DOI:** 10.1016/j.jpi.2024.100393

**Published:** 2024-08-05

**Authors:** Aleksandar Vodovnik

**Affiliations:** Department of Pathology, Førde Central Hospital, Førde, Norway

To Editor

We have fully digitized our pathology department in 2013. As a part of our digital pathology project, 10 validation studies, covering all aspects of our pathology workload, have been performed, yielding multiple publications, one of which showing a shorter digital diagnostic time, compared to the microscopy.^1^, ^2^, ^3^ We have since actively observed multiple tasks affecting a non-diagnostic time. Objective measurements of a non-diagnostic time were deemed logistically unfeasible for one person standing for diagnostics and measurement and, therefore, an empirical study was performed. The main reasons were obvious problems with execution of measurements (extensive stop-and-go tasks, multiple times throughout the biopsy reading process), standardization of measurements (differences in approach to diagnostics of different biopsies), and difficulties to repeat the same non-diagnostic tasks a second time, for comparison. A total diagnostic time has been defined as a sum of a diagnostic time (directly related to diagnosis) and a non-diagnostic time (crucial but not directly related to diagnosis). Our mixed clinical workload, including diagnostic biopsies, resections, autopsies, and non-gynecological and fine needle aspiration cytology, has substantially changed over the years. Between 2013 and 2023, an absolute number of patient entries increased by 36%, and number of slides by 86%, largely regarding diagnostic biopsies and resections.

Slide handling (putting on microscope, moving around, returning to tray, checking up against request form for demographics, and gross description), image adjustment, magnification exchange, slide annotations and measurements, report preparation, medical coding, and sign-off were non-diagnostic tasks observed between the microscopy and digital pathology platforms on 14″ laptop (Elite Book 840, Hewlett Packard) by the senior pathologist, working mainly from the home office. We used a Nikon Eclipse i80 for microscopic diagnosis before. Over the period of 10 years, 3 digital platforms for routine reporting were set up: laboratory information management system (LIMS) SymPathy (Tieto) integrated with Aperio AT Turbo scanner (Leica); Unilab LIMS (Alphasoft) integrated with Aperio AT Turbo scanner; and Unilab LIMS integrated with Sectra IDS7 and Aperio GT Turbo 450 Dx scanner. An increased productivity (number of cases/slides reported per hour) served as a measure of potential savings in a digital non-diagnostic time. The data were retrieved from our LIMS.

Slide handling-related tasks were all consolidated and faster in digital pathology. Responsibilities for physical slide handling were completely transferred to the laboratory staff. The whole system was built around the bar coding, following patient's samples from the clinic to the pathology department and throughout. As a result, any discrepancies in data between the slide and the patient were picked up by the system and dealt with by the laboratory staff. There was, therefore, no need for any slide handling related tasks by pathologists as they were all consolidated on the digital platform. Image adjustment, magnification exchange and slide annotations and measurements (time used for regarded as technically non-diagnostic) were all faster in a digital pathology. A fine calibration of image quality was not possible on the microscope and was regarded as a very useful, time-saving (lower rescanning rates) digital feature, although artificial. In addition, continuous digital magnifications (up to 40×) were superior to the fixed magnifications on the microscope. Sliding bar/double click digital functionalities are almost immediate and digital images are expected to automatically appear in focus, with only occasional cases rescanned in our hands, due to sample scarcity and minor technical difficulties (under 0.1% slides). Annotations and measurements were simpler to perform, more consistent and permanent by choice in digital pathology. Report preparation, coding, and sign-off were consolidated and faster on digital pathology platform, supported by canned reports and a voice recognition interface. By putting in a disease code, we can generate a complete report for normal samples and most common entities (canned reports), ready for sign-off immediately after. Synchronized viewing of multiple images and double click for 0–40× zoom functionalities in Sectra were crucial to the faster digital diagnostics, as not available on the microscope. The one-step login access to our digital platform contributed to the faster operation of all non-diagnostic tasks, as well. All images from radiology, endoscopy, and dermatology departments are made available on our digital platform, contributing to the faster turnaround time. The workload data showed an increase in productivity by 30%, through savings in a digital non-diagnostic time, compared to the microscope. A non-diagnostic time could take up to 90% of total diagnostic time in unchallenging lesions, which amount to the considerable portion of the routine pathologist workload. Depending on the departmental workload type, this could yield even higher savings in a digital non-diagnostic time, and subsequently lead to the higher increase in the productivity.

The one-step login with consolidations of multiple tasks in digital pathology and system innovations exclusive to digital pathology have led to the considerable decrease in a non-diagnostic time. In our experience, shorter diagnostic and non-diagnostic digital time together amounted to an increased productivity by 40%, compared to microscopy. This time saving could be used to cover increased workload and its complexity, compensate for staffing shortages and absences, and other departmental activities (teaching, research, lab improvements etc.), all to the benefit of our patients. We are aware of the differences between pathology departments worldwide in type, size, workload, organization, staffing etc., which may potentially and variably skew observed data. But the bottom line is that the digital pathology brings multiple and valuable professional, health, and financial benefits.^3^, ^4^, ^5^

## Declaration of competing interest

The authors declare that they have no known competing financial interests or personal relationships that could have appeared to influence the work reported in this paper.
